# Combined argon laser peripheral iridoplasty and Nd: YAG laser shock wave therapy for recurrent XEN gel stent obstruction due to iris incarceration: A case report

**DOI:** 10.1097/MD.0000000000026652

**Published:** 2021-07-23

**Authors:** Seungsoo Rho, Su-Ho Lim

**Affiliations:** a Department of Ophthalmology, CHA Bundang Medical Center, CHA University, Seongnam, South Korea; b Department of Ophthalmology, Daegu Veterans Health Service Medical Center, Daegu, South Korea.

**Keywords:** argon laser iridoplasty, glaucoma, iris incarceration, neodymium-doped yttrium aluminum garnet laser, XEN gel stent

## Abstract

**Introduction:**

Despite its proven effectiveness and safety profile, the XEN Gel Stent (Allergan Inc., CA, USA) has a small lumen and is therefore likely to become occluded by fibrin, a blood clot, or even the iris. However, few studies have investigated XEN-iris occlusion and how to manage this condition. We describe the first case report of recurrent XEN gel stent obstruction by iris incarceration, which was resolved following a combined treatment with argon laser peripheral iridoplasty (ALPI) and low-energy neodymium-doped yttrium aluminum garnet (Nd: YAG) laser shock wave treatment.

**Patient information:**

A 74-year-old Korean male underwent uncomplicated XEN gel stent implantation and presented with low intraocular pressure (IOP) with a well-functioning filtering bleb during the first postoperative week. On postoperative day 10, the XEN lumen was occluded by the iris and demonstrated an IOP spike of 33 mmHg. Despite the use of pilocarpine, the iris incarceration persisted. Therefore, surgery to reposition the XEN stent was attempted using a gonio-prism and intraocular forceps. After the first revision surgery, the IOP and stent position were stable for 2 weeks. However, recurrent partial obstruction of the stent by the iris, pigment dispersion into the intraluminal space, and an elevated IOP of 24 mmHg were observed later.

**Diagnosis:**

Recurrent XEN gel stent occlusion by the iris and intraluminal pigment dispersion.

**Interventions:**

Combined ALPI and low energy Nd: YAG laser shock wave therapy.

**Outcomes:**

IOP dropped from 24 mmHg to 10 mmHg immediately and continued to be well-controlled until 3 months later (range: 8–12 mmHg).

**Conclusions:**

To the best of our knowledge, this is the first case report of the efficacy of combined laser treatment for relieving recurrent XEN implant occlusion by the iris. This combination laser treatment might be a relatively safe rescue treatment to restore the patency of a XEN gel stent occluded by the iris, even in cases with recurrent XEN stent obstruction after surgical repositioning.

## 1 Introduction

Glaucoma is the leading cause of irreversible vision loss globally.^[1]^ Surgical intervention for glaucoma treatment is usually required when medical or laser procedures have been used, consider to be failed in advanced disease stage.^[1,2]^ The XEN gel stent (Allergan Inc., CA, USA) is a 6-mm-long hydrophilic tube made of collagen-derived gelatin cross-linked with glutaraldehyde.^[3]^ The XEN implant decreases intraocular pressure (IOP) by creating a permanent drainage shunt from the anterior chamber (AC) to the subconjunctival space through a scleral channel; many previous studies have demonstrated a reasonable IOP-lowering effect.^[4]^ To minimize the risk of postoperative hypotony, the internal diameter is designated as 45 μm.^[3,4]^ Despite its proven effectiveness and safety profile, the XEN gel stent may still become occluded by fibrin, a blood clot, or the iris due to its design with a small internal lumen.^[5–8]^

To date, few studies have explored XEN-iris occlusion and how it should be managed.^[5,8]^ Herein, we describe the first case report of a recurrent XEN gel stent obstruction by a “plug” shaped iris incarceration, which effectively resolved after combined treatment with argon laser peripheral iridoplasty (ALPI) and low energy neodymium-doped yttrium aluminum garnet (Nd: YAG) laser shock wave treatment.

## 2 Case report

This study adhered to the principles of the Declaration of Helsinki. Written informed consent for the report and photographs were obtained from the patient. This case study was approved by the Institutional Review Board of Daegu Veterans Health Service Medical Center.

### 2.1 Initial presentation

**Figure 1:**
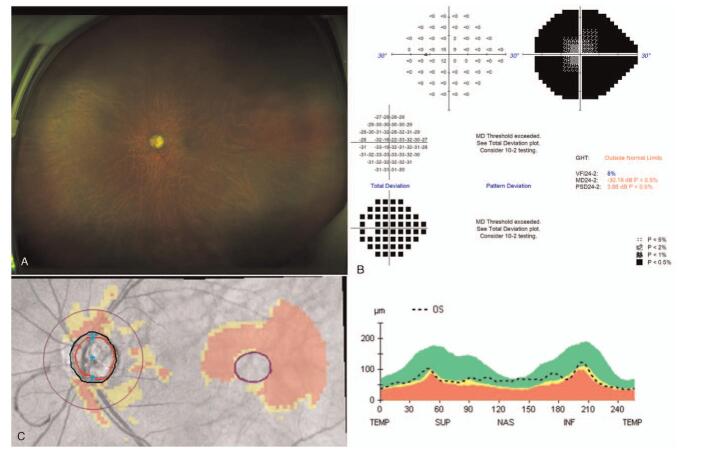
The subject's preoperative ultra-widefield fundus photograph, visual field test, and optical coherence tomography analysis.

A 74-year-old Korean male was referred to the Daegu Veterans Health Service Medical Center for progressive loss of vision in his left eye. The patient had been diagnosed with advanced primary open-angle glaucoma. Two years prior to presentation, the patient had undergone uneventful cataract surgery in the left eye at another hospital. He had been receiving maximal medical treatment, including a combination of dorzolamide and timolol (Cosopt, Santen, Japan), 0.15% brimonidine tartrate (Alphagan, Allergan, USA), latanoprost (Xalatan, Pfizer, USA), and 250 mg acetazolamide 3 times daily. At the time of presentation, his best-corrected visual acuity in the left eye was 20/200, and the IOP in the same eye was 24 mmHg. Slit lamp examination revealed a deep and quiet AC and a well-positioned intraocular lens. The cup-to-disc ratio was 0.95, with superior and inferior notching, diffuse retinal nerve fiber layer (RNFL) thinning, and a slightly pale optic disc in the left eye. Visual field testing revealed tunnel vision (mean deviation: - 30.18 dB, visual field index: 8%). Spectral domain optical coherence tomography also demonstrated a generally reduced RNFL thickness (Fig. 1). Gonioscopy showed a wide iridocorneal angle (D40r, Spaeth classification). Brain magnetic resonance imaging (MRI) findings were also unremarkable.

### 2.2 The surgical procedure for XEN gel stent implantation

The XEN gel stent was implanted *ab interno* in the usual manner. Briefly, under topical anesthesia, 0.1 mL mitomycin C, MMC (0.2 mg/mL) was injected with a 30-gauge needle in the supero-nasal quadrant. The intended area of placement in the supero-nasal quadrant, which was 3 mm from the limbus, had previously been marked. Next, the *ab-interno* XEN gel stent was implanted using a mirrored goniolens following the rule of 1 mm in the AC, 2 mm tunneled through the sclera, and 3 mm in the subconjunctival space. The injector was withdrawn gently, and the optimal XEN placement was confirmed. Subsequently, the viscoelastic material was removed from the AC. Finally, we checked the bleb formation and its function with the forced infusion of a balanced salt solution. The patient was started on levofloxacin and prednisolone acetate 4 times daily on the next day.

### 2.3 XEN occlusion by iris incarceration and surgical repositioning

**Figure 2:**
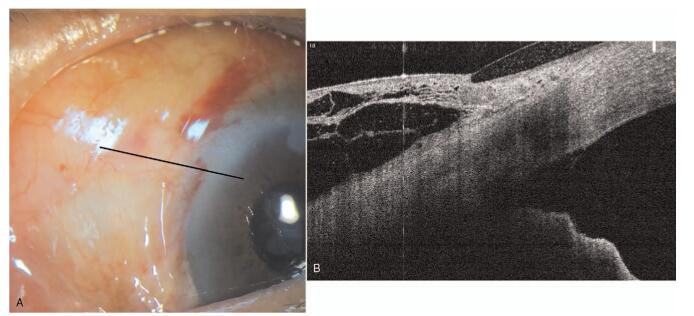
Anterior segment photography and optical coherence tomography demonstrated a relatively well-functioning filtering bleb 5 days after the XEN implantation. Black line indicates scan line of optical coherence tomography,

**Figure 3:**
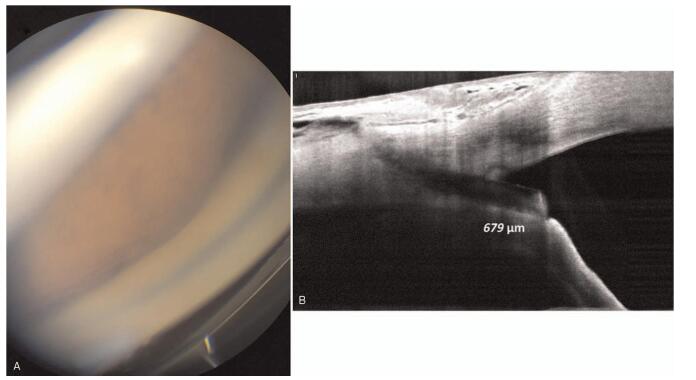
Gonioscopic examination and anterior segment optical coherence tomography revealed the iris incarceration into XEN gel stent. The length of XEN implant in the anterior chamber was 679 μm.

The IOP was 6 mmHg on day 1 and 8 mmHg on day 5 with a well-functioning bleb (Fig. 2A and B). However, the patient's IOP spiked to 33 mmHg on postoperative day 10. Gonioscopic examination showed that the tip of the implant was not visible and resembled peripheral anterior synechiae (Fig. 3A). Anterior segment optical coherence tomography (AS-OCT) examination revealed that the XEN implant was occluded by iris incarceration. The length of the XEN implant protruding into the AC was 679 μm (Fig. 3B).

Topical anti-glaucoma treatment was restarted (dorzolamide/timolol combination), and 2% pilocarpine (Isopt Carpine, Alcon, USA) was added in an attempt to relieve the obstruction with miosis. Despite these attempts, the iris occlusion did not resolve. Therefore, the author planned a repositioning surgery.

**Figure 4:**
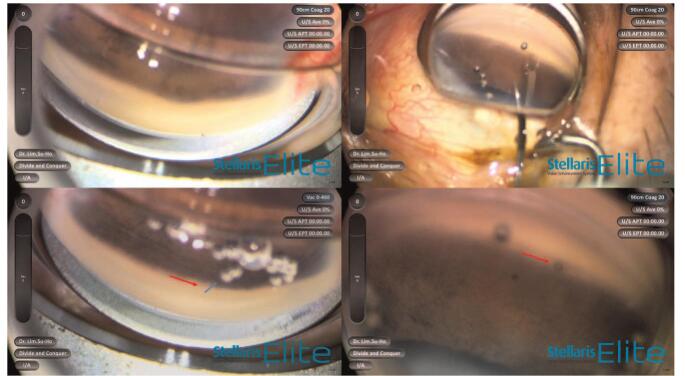
Intraoperative findings for XEN repositioning surgery under a surgical microscope. Red arrow indicates XEN gel stent. Blue line indicates protruded portion of XEN.

Under the surgical microscope, an ophthalmic viscoelastic device (1.4% sodium hyaluronate) was injected via a side port incision to deepen the AC and to release the iris incarceration. We identified the XEN implant using a modified Swan-Jacob surgical gonioprism. The XEN implant was repositioned into the AC using a 25-gauge long intraocular forceps (single-use serrated vitreoretinal ILM forceps). To ensure the flow through the implant, the eye was pressurized with a balanced salt solution, and a relatively well-elevated conjunctival bleb was noted. A bandage contact lens was placed at the end of the surgery to prevent transient hypotony (Fig. 4).

**Figure 5:**
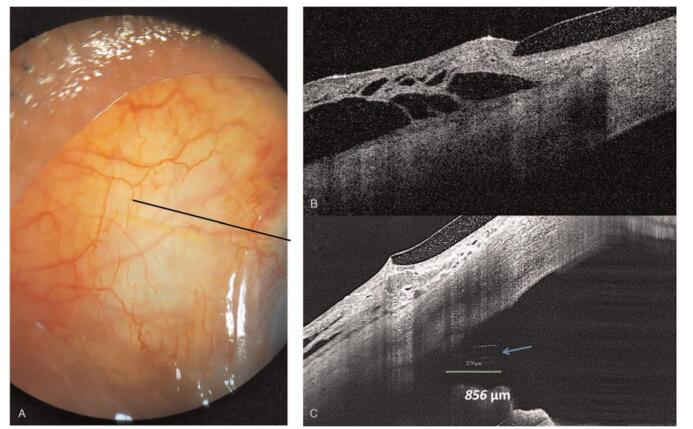
One day after the revision surgery, anterior segment photography and an anterior segment optical coherence tomography examination demonstrated re-canalization of the internal ostium XEN gel stent and release of the iris incarceration (Blue arrow). The length of XEN in the anterior chamber was 856 μm (Green line). Black line indicates the scan line of optical coherence tomography.

One day after the revision surgery, the IOP was 6 mmHg, and a relatively well-positioned tube (approximate length of the protruding tube: 856 μm in the AC) was observed on AS-OCT examination. During the first 2 weeks after the revision surgery, the IOP was stable between 6 and 12 mmHg (Fig. 5).

### 2.4 Second XEN gel stent partial occlusion, and argon laser peripheral iridoplasty

**Figure 6:**
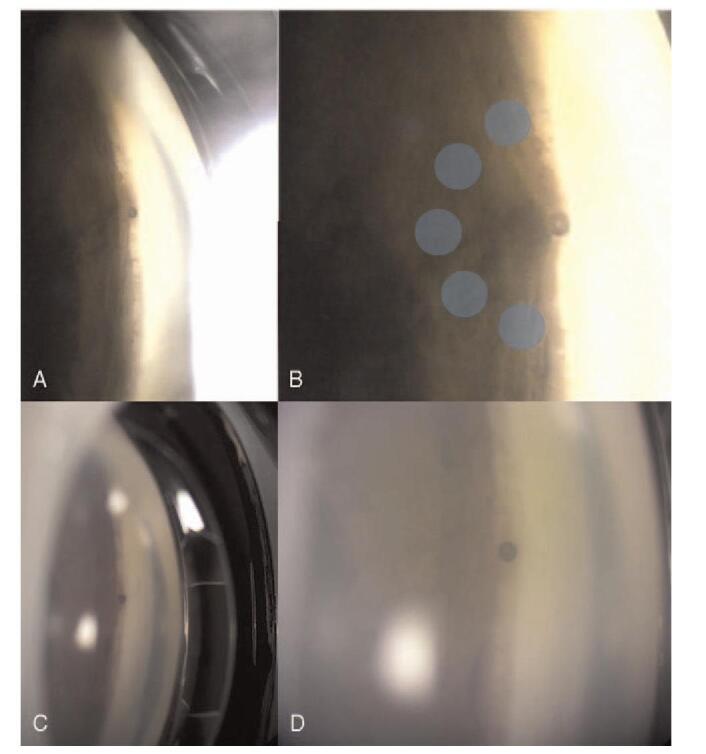
The gonioscopic examination both before and after the combined treatment with Argon laser peripheral iridoplasty and Nd: YAG shock wave therapy of the peri-luminal area. A total of five shots (500 μm, 260 m/W, 500 m/sec) were made in a C-shaped configuration surrounding the occluded XEN implant tip to retract the tissue against the flow into the XEN tip (Blue circles).

Two weeks after the repositioning surgery, the patient visited our facility again because of blurred vision. His IOP was 24 mmHg, with a slightly low bleb height with no medication. Gonioscopic examination revealed that the internal ostium of the XEN gel stent was partially occluded by the iris, and mild pigment dispersion was visible into the intraluminal area (Fig. 6A and B).

ALPI was attempted as a treatment option to liberate the iris occlusion and re-establish flow. A total of 5 shots (500 μm, 260 m/W, 500 m/sec) were made in a C-shaped configuration surrounding the occluded XEN implant tip to retract the tissue against the flow into the XEN tip. (Fig. 6B) After ALPI, the iris incarceration was resolved. Additional low energy neodymium-doped yttrium aluminum garnet (Nd: YAG) laser shockwave treatment for with peri-luminal AC tip was applied to improve flow (0.8 mJ, 2 shots). After treatment, the internal ostium of the XEN was clearly visible. (Figs. 6C and D).

## 3 Results

The IOP of 2 hours after the ALPI and Nd: YAG laser treatment was substantially reduced to 10 mmHg. One week later, the patient returned with a functioning bleb and a patent XEN gel stent. Gonioscopic examination showed that the tip of the implant was not obstructed and was surrounded by a laser mark on the iris. AS-OCT examination also revealed a patent XEN gel stent opening. His IOP was 12 mmHg and continued to be well-controlled until 3 months later (range: 8–12 mmHg)

## 4 Discussion

The XEN45 gel stent has been recognized as a safe and effective minimally invasive glaucoma surgery procedure even in advanced and refractory glaucoma.^[3]^ This XEN stent has been designated to have small diameter of 45 μm to prevent early postoperative hypotony.^[3,4]^ In contrast, the XEN gel stent also carries a risk of occlusion by fibrin, a blood clot, or the iris due to its small internal lumen.^[5–8]^ A relatively shallow AC and a crowded anterior segment in the Asian eye might increase the possibility of anatomical obstruction of the XEN implant by the iris. In these contexts, Sng et al estimated a 3.2% risk of developing XEN stent iris occlusion.^[9]^ However, there are limited reports of XEN iris occlusion and its management in the literature.^[5,8]^

In our patient, the first iris occlusion was observed on postoperative day 10. Similarly, Tadrosse and Khouri also reported XEN iris occlusion at 10 days after surgery.^[8]^ However, in our patient, the internal ostium of the XEN gel stent was not observed on office-based gonioscopy. Thus, we decided to attempt revision surgery to identify and reposition the implant, if needed. Although intraoperative gonioscopy confirmed the positioning of the implant and the release of iris incarceration during the revision surgery, the tip of the stent was partially re-occluded by the iris 2 weeks later.

Possible explanations for the first occlusion are as follows:

1. a slightly low (posterior) position of the XEN gel implant,
2. the relatively short length of the protruded implant into the AC,
3. excessive filtration and a local turbulence effect in the early postoperative phase,
4. rubbing the eye with the hands of the patient, and
5. floppy nature of the subject's iris.

In this patient, the gonioscopic examination revealed a slightly lower position of the XEN gel implant, which could have resulted in steepening between the implant and the iris root. Recently, Rafael et al reported that a more posterior stent placement was associated with an increased rate of early complications.^[10]^ The relatively short length of 679 μm and the presence of early postoperative hypotony-induced AC shallowing together might bring the iris closer to the stent tip.^[8]^ Moreover, an impaired outflow function via the trabecular meshwork, which is typically seen in advanced glaucoma, might have made the fluid dynamics to become more dependent on the XEN gel stent. Based on the AS-OCT examination, we thought that all these mechanisms might induce “plug-in” shape incarceration and secondary iris steepening of the surrounding iris that resembled peripheral anterior synechiae.

Surgical revision was only able to resolve the possible mechanisms for the relatively short length of the protruded implant into the AC. Other factors may have led to the recurrent partial occlusion of the XEN gel stent. Argon laser iridoplasty can induce thermal-induced iris contracture. This contracture resulted in

1. distancing the iris from the stent and
2. a new wider configuration of the peripheral angle itself by minimizing irido-corneal contact.

Finally, the laser procedure resolved the occlusion and restored the flow through the XEN gel stent. Our favorable result was also supported by Tadrosse and Khouri.^[8]^

In our patient, mild pigment dispersion into the intraluminar portion of the XEN implant was also observed. Eagle and Razeghinejad reported XEN occlusion with a pigmented epithelium early in the postoperative period.^[5]^ In addition, Scantling-Birch et al suggested that YAG-laser fibrinolysis could lead to successful clearing of lumen obstructions.^[11]^ On the basis of these previous reports,^[5,11]^ we also performed the peri-lumen Nd: YAG shock wave treatment for re-canalization. As a result, we were able to re-establish the flow of the iris-occluded XEN implant with this combined laser treatment.

## 5 Conclusions and clinical significance

To the best of our knowledge, this is the first case report of the efficacy of combined laser treatment (ALPI and Nd: YAG laser shock wave) for recurrent XEN implant occlusion by the iris. ALPI and Nd: YAG laser shock wave therapy might be a relatively safe rescue treatment for XEN iris occlusion, even in cases with a history of previous surgical repositioning of the XEN implant.

## Acknowledgments

The authors wish to thank eWorldEditing and Paperpal Preflight for language editing.

## Author contributions

**Conceptualization:** Seungsoo Rho, Su-Ho Lim.

**Data curation:** Seungsoo Rho, Su-Ho Lim.

**Formal analysis:** Seungsoo Rho, Su-Ho Lim.

**Funding acquisition:** Su-Ho Lim.

**Resources:** Su-Ho Lim.

**Writing – original draft:** Seungsoo Rho, Su-Ho Lim.

**Writing – review & editing:** Seungsoo Rho, Su-Ho Lim.
